# Attachment Patterns and Complex Trauma in a Sample of Adults Diagnosed with Gender Dysphoria

**DOI:** 10.3389/fpsyg.2018.00060

**Published:** 2018-02-01

**Authors:** Guido Giovanardi, Roberto Vitelli, Carola Maggiora Vergano, Alexandro Fortunato, Luca Chianura, Vittorio Lingiardi, Anna Maria Speranza

**Affiliations:** ^1^Dipartimento di Psicologia Dinamica e Clinica, Facoltà di Medicina e Psicologia, Sapienza Università di Roma, Rome, Italy; ^2^Dipartimento di Neuroscienze e Scienze Riproduttive ed Odontostomatologiche, Università degli Studi di Napoli Federico II, Napoli, Italy; ^3^Ospedale San Camillo, Rome, Italy

**Keywords:** gender dysphoria, transgender, clinical health psychology, attachment, complex trauma, parental relationships

## Abstract

The current study investigated attachment representations and complex trauma in a sample of gender dysphoric adults. Although it has been proven that the psychological wellbeing of gender diverse persons is largely mediated by family acceptance and support, research on their relationships with parental figures is scarce. A total of 95 adults took part in the study. The attachment distribution was as follows: 27% secure, 27% insecure and 46% disorganized. Regarding early traumas, 56% experienced four or more traumatic forms. Further, gender dysphoric adults showed significantly higher levels of attachment disorganization and polyvictimisation, relative to controls. Comparisons of subgroups, defined by natal gender, showed that *trans* women, compared to control males, had more involving and physically and psychologically abusive fathers, and were more often separated from their mothers; *trans* men, relative to female controls, had more involving mothers and were more frequently separated from and neglected by their fathers. The research has several implications for treatment, clinical health psychology, family support and education.

## Introduction

Gender dysphoria (GD) is described in the *DSM-5* (American Psychiatric Association [APA], 2013) as a condition in which individuals experience distress due to incongruence between their gender identity (or experienced/expressed gender) and the gender they were assigned at birth. Findings about the incidence and prevalence of this condition are controversial (Coleman et al., 2012). De Cuypere et al. (2007), in their review involving eight countries, found a prevalence ranging from 1:11,900 to 1:45,000 for *trans* women^1^ and 1:30,400 to 1:200,000 for *trans* men. Some authors have suggested even higher – or increasing – prevalence rates (e.g., Olyslager and Conway, 2007; Collin et al., 2016; for a review, see Arcelus et al., 2015), which are supported by clinical observations (Cole et al., 1997; Coleman et al., 2012). With regard to etiology, no unequivocal factor has been found to determine GD (Meyer-Bahlburg, 2009; de Vries and Cohen-Kettenis, 2012). Rather, GD is widely considered a multi-factorial condition in which both psychosocial and biological aspects play a role (de Vries and Cohen-Kettenis, 2012; American Psychiatric Association [APA], 2013). The onset of GD typically occurs very early, around the ages of 2 through 4, although it is also possible for the onset to occur much later, during adolescence or even adulthood (Nieder et al., 2011; American Psychiatric Association [APA], 2013; Zucker et al., 2016). Many children who experience GD do not continue to experience it after puberty. Studies have found the percentage of persistence into adolescence and adulthood to vary from 2 to 20% (Steensma et al., 2011, 2013). While factors associated with the persistence of childhood GD are still unknown, the intensity of GD seems to be an important predictor of persistence after puberty (Steensma et al., 2013). Whether or not their GD persists, gender diverse children and adolescents, compared to no-GD youth, are at greater risk of suffering many psychological and social adversities. They experience poorer peer relations (e.g., Cohen-Kettenis et al., 2003; Zucker et al., 2012) and poorer family relations (e.g., Grossman and D’Augelli, 2006; Hill and Menvielle, 2009), and they are more psychologically vulnerable (e.g., Cohen-Kettenis et al., 2003; Singh et al., 2011; Steensma et al., 2014). Further, their psychological problems seem to be of a more internalized nature (e.g., presenting in depression and anxiety) than an externalized nature (Coates and Person, 1985; Rekers and Morey, 1989; Zucker and Bradley, 1995; Cohen-Kettenis et al., 2003; Steensma et al., 2014). However, there has been considerable variability in the findings. For instance, percentages of clinical-range cases reported in studies using the Child Behavior Checklist total behavior problem score range from 13 to 84% (for an overview, see Zucker et al., 2014). As Ristori and Steensma (2016) point out, to better understand the association between GD and the variability of psychological functioning in children and adolescents, we should consider the effect to be largely mediated by social intolerance to gender diversity. Variability in psychological functioning in children with GD is expected to be inversely correlated with the intensity of their experienced social intolerance. Accordingly, there is evidence that perception of support within the family and with friends reduces the degree of psychosocial impairment (e.g., Simons et al., 2013) and thus functions as a protective factor (Shiffman et al., 2016). However, research on the family environment and relations of children and adolescents with GD is scarce. As Dierckx et al. (2016) underline, research of this kind tends to be more qualitative than quantitative. Negative reactions from parents, and sometimes even their rejection of the child, is mentioned in various articles (Di Ceglie and Thümmel, 2006; Hill et al., 2010; Riley et al., 2011), but few studies have tried to explore the parental relationships and the traumatic experiences that can occur in the family environment of a child or adolescent with GD.

In this study, we evaluated the attachment patterns and history of early relational traumas in a sample of *trans* adults with early onset GD (first childhood) (American Psychiatric Association [APA], 2013), in order to describe the less studied risk factors for the psychological outcomes of GD youth. Considering the variety of life paths that can lead to a GD diagnosis, and following the recent directions taken by the trauma literature, we conceptualized trauma more broadly than the definition expressed by PTSD diagnosis. We drew on the concept of “complex trauma” (Courtois, 2004; Cook et al., 2005), which includes child maltreatment, neglect, domestic violence and other attachment-related trauma (Bailey et al., 2007), in addition to PTSD. Given that complex trauma and GD are both multi-determined constructs, we thought it more profitable to conceive of early onset GD and early relational traumas as concurring risk factors for adversity in psychological development. In this framework, we considered trauma a dimension independent of GD, and treated traumatic experiences in primary relationships – drawn close to attachment patterns – as important features of the complexity presented by gender variant individuals, rather than causative factors of their GD, as previously suggested in the literature (see below).

### Gender Dysphoria and Complex Trauma

Complex trauma can be understood as a set of experiences of cumulative, chronic and prolonged traumatic events, most often of an interpersonal nature, involving primary caregivers and frequently arising in early childhood or adolescence (Courtois, 2004; Cook et al., 2005). Maltreatment experiences may include: severe neglect; exposure to domestic violence; intensive, painful medical conditions; and physical and sexual abuse (Zilberstein, 2014). Often, children suffering from complex trauma face a combination of these experiences (Ford et al., 2010). Such children are at risk of developing disorganized attachment relationships in infancy. The percentage of disorganized attachments in high-risk families has been shown to range from about 40 to 80% (Lyons-Ruth and Jacobvitz, 2008; Liotti, 2013). The environment provided by maltreating parents usually induces children to develop dissociative and avoidant strategies to cope with extreme emotions (Solomon and George, 1999; Briere, 2006; Cicchetti and Valentino, 2006; Bailey et al., 2007), and this prevents them from processing and normatively integrating memories and experiences (Fonagy et al., 2000; Macfie et al., 2001).

The relationship between relational trauma and GD has often been described as univocal. Many authors (e.g., Money and Lamacz, 1984; Stoller, 1985; Money, 1986; Zucker and Kuksis, 1990; Marantz and Coates, 1991; Zucker and Bradley, 1995) have speculated about the influence of trauma, abuse, dysfunctional parental conduct (such as a mother’s extreme closeness with her child), parental dynamics or pathology (such as maternal depression or the absence of a father) and parents’ atypical psychosexual development (such as their confusion about their own feelings of masculinity and femininity) on children. It has been thought that such environmental patterns might limit children’s opportunities to identify with the same-sex parent and to experience cross-gender reinforcement patterns (for a discussion, see Hill and Menvielle, 2009). Transsexualism has often been interpreted as an extreme dissociative defense against trauma experienced in early relationships (Coates and Person, 1985; Devor, 1994; Di Ceglie, 1998). However, to date, no solid empirical support has been produced by studies testing these hypotheses. The co-occurrence with dissociation, for instance, suggested by many theoretical and clinical studies (e.g., Coons, 1984; Putnam, 1989; Devor, 1994; Modestin and Ebner, 1995), was recently effectively disputed (Colizzi et al., 2015).

As mentioned above, research on the experience of complex trauma in GD individuals is limited. In comparison to the general population, individuals with GD have been shown to be more subject to devaluation, oppression and discrimination (e.g., Lombardi et al., 2002; Melendez and Pinto, 2007; Bockting et al., 2013). Some (mostly descriptive) studies have reported a high frequency in transsexual individuals of childhood sexual and physical abuse perpetrated by parents and caregivers (Pauly, 1974; Lothstein, 1983; Devor, 1994; Gehring and Knudson, 2005; Wilchins, 2006; Wharton, 2007; Veale et al., 2008; Cussino et al., 2017; Lingiardi et al., 2017). Moreover, one study showed that patients in gender clinics were more likely than psychiatric patients to report parental death during adolescence and early adulthood (Bernstein et al., 1981). As Bandini et al. (2011) point out, children showing gender atypicality are at higher risk of maltreatment and abuse, and suffer reprisals from parents as a consequence of their cross-gender behavior (Corliss et al., 2002; Nuttbrock et al., 2010). Nuttbrock et al. (2009), after interviewing a wide sample of *trans* women, reported that the majority of the women had failed to disclose their gender incongruence to parents and siblings in early adolescence, and thus had been unable to benefit from the protection of family support, which has been shown to be a protective factor for mental health (Simons et al., 2013). Three other studies with *trans* samples (Grossman et al., 2005, 2006; Grossman and D’Augelli, 2006; Grossman et al., 2006) showed that many GD youth reported experiences of verbal and physical abuse from family members and neighbors, and negative reactions from parents when they came out.

### Gender Dysphoria and Attachment

Few studies on GD patients have examined attachment processes and patterns. With regard to children, Goldberg (1997) reported a higher rate of insecurity in boys with GD (formerly known as “gender identity disorder” [GID]), compared to a normative population. Birkenfeld-Adams (2000) confirmed this higher percentage of boys with GID in a modified Strange Situation, finding insecure attachment in 73% of the sample. Cooper et al. (2013) highlighted a negative association between the related construct of “gender contentedness” and avoidant attachment in both boys and girls, and with preoccupied (ambivalent) attachment in boys in a fifth grade sample. As DeKlyen and Greenberg (2016) state, youth with GD seem to be at increased risk for insecure attachment; however, there is no consensus on the form of this insecurity.

Few researchers have used the Adult Attachment Interview (AAI) with transsexual or gender-variant populations. Cook (1999) studied attachment patterns in a group of six fathers of GID boys, finding the presence of four unresolved/disorganized states of mind and two that were preoccupied with a secondary unresolved classification. Vitelli and Riccardi (2010) found, in a sample of 18 transsexual adults, a high percentage of unresolved/disorganized states of mind (39%) and insecure patterns (72%). The authors also found an impressively high frequency of traumatic experiences within the first 10 years of life. Colizzi et al. (2013) found a high percentage of insecure attachment (70%) in their sample of 50 adult transsexuals. Finally, in a recent study, Lingiardi et al. (2017) found a high percentage of unresolved/disorganized states of mind (50%) and a considerably high percentage of secure patterns (39%). Moreover, 29 (66%) of the 44 individuals had traumatic childhood experiences relating to the loss of an attachment figure or physical or sexual abuse.

In response to the identified gaps in the literature, this study aimed at gaining in-depth knowledge of some problematic features that may be grounds for psychological complications in *trans* people and may interfere with their gender expression. Specifically, we aimed at exploring gender variant persons’ attachment representations and history with primary figures in early experiences of trauma and loving caregiving, and comparing these findings with those of a cisgender control group. In order to explore the internalized aspects of parental relationships in our subjects, we narrowed our analysis to the Adult Attachment Interview subscales concerning states of mind directly connected to parental figures (e.g., idealization, anger and derogation). At a deeper level of enquiry, we then performed comparisons of subgroups. To analyze attachment and trauma, we broke our sample down according to gender (*trans* women vs. *trans* men). Additionally, regarding trauma, we compared *trans* women to male controls and *trans* men to female controls, in order to explore the differences in natal males and females between transgender and cisgender developmental paths. At the heart of our research was an intention to update psychological and clinical perspectives on the developmental environment of *trans* persons, highlighting the impact that adversities in the family context may have on their adult adaptation. Our aim was to read these adversities not as they have been read in the past – as etiological factors for GD – but as instances of risk that should be carefully managed by psychologists working with *trans* people. Ultimately, the purpose of this study was to contribute to and facilitate *trans* persons’ development of a free and personal identity during their transition path.

To sum up, we aimed to test the following hypotheses:

(1) The first hypothesis postulated that in our sample of gender dysphoric people unsecure and disorganized attachment classifications were more frequent, relative to controls;
(2) The second hypothesis assumed that in the gender dysphoric sample higher frequencies of traumatic experiences could be found within early caregiving relationships, compared to controls;
(3) The third hypothesis postulated that in the gender dysphoric sample early experiences of a loving caregiving were less frequent compared to controls;
(4) The forth hypothesis assumed that it was possible to find differences regarding attachment and complex trauma between *trans* women and *trans* men.

## Materials and Methods

### Participants

The study was conducted with 95 GD adults (74 *trans* women, 21 *trans* men). The sample was recruited from the Service for Adaptation of Physical Identity to Psychological Identity Clinical Center (SAIFIP, San Camillo-Forlanini Hospital, Rome) and the Gender Dysphoria Psychological Service – Department of Clinical Neurosciences, Reproductive and Odontostomatological Sciences (UPCPA, Federico II University Hospital, Naples). All participants declared an early onset of GD (first childhood). The mean age of the full sample was 29 years (*SD* = 7.2); the same mean age was identified for *trans* women (*SD* = 7.8) and *trans* men (*SD* = 5.0). All *trans* men were Italian, while 86% of *trans* women were Italian and 14% were South American. With respect to the level of education, 3% had an elementary license, 39% had a middle school license, 51% had a high school diploma and 7% had a college degree.

The control group, which was recruited through snowball sampling by the authors in Rome and Naples, was composed of 123 non-clinical persons (61 male, 62 female). The mean age of the total sample was 34 years (*SD* = 10.2); the mean age of males was 35 years (*SD* = 11.9) and the mean age of females was 33 years (*SD* = 5.6). Aside from 2% of the sample, all were Italian. With respect to the level of education, 27% had a middle school license, 54% had a high school diploma and 19% had a college degree. The overall sample was recruited expressively asking for a participation secured through a written informed consent procedure that required active consent from participants. The current study has been attended within the Ph.D. context at the Department of Dynamic and Clinical Psychology (Faculty of Medicine and Psychology, Sapienza University of Rome) and received the approval from the Ethical Committee of the Department (n° 24/2016).

### Measures

#### Adult Attachment Interview (AAI; George et al., 1984; Main et al., 2003)

The AAI is a semi-structured interview following an hour-long protocol, which explores adults’ mental representations of attachment while discussing childhood experiences. After providing an overview of their family composition, respondents are asked to describe their early relationships with parents, supplying five adjectives and supporting these with descriptions of specific incidents. Additionally, interviewees are probed regarding situations of distress (e.g., emotional upset, hurt, or illness) and instances of threat or abuse. Following this, questions are posed concerning respondents’ early reactions to separation from a caregiver and significant loss. The interview transcripts are scored on thirteen 9-point scales. Five scales assess the “probable childhood experience” with caregiving figures (loving, rejecting, neglecting, involving, pushing to achieve), whereas eight scales assess the current mental state with regard to attachment (idealization, anger, derogation, passivity, lack of memory, meta-cognition, coherence of transcript, and coherence of mind). The AAI score is based on the participant’s ability to produce coherent narratives of childhood experiences with caregivers that classify the respondent as Secure/Autonomous (F), Dismissing (Ds), Entangled/Preoccupied with respect to attachment (E) or “Cannot Classify” (CC) when a global breakdown in the organization of discourse arises. An interviewee may also be assigned an Unresolved/Disorganized state of mind (U) concerning past abuse or loss in association with a best-fitting primary classification. Psychometric testing and meta-analyses have provided evidence of the AAI’s stability and discriminant and predictive validity in both clinical and non-clinical populations (Bakermans-Kranenburg and Van IJzendoorn, 1993; Bakermans-Kranenburg and van IJzendoorn, 2009; Sagi et al., 1994; van IJzendoorn, 1995; Roisman et al., 2007; Hesse, 2008; van IJzendoorn and Bakermans-Kranenburg, 2008). In the present study, each interview was recorded, transcribed verbatim and coded by certified raters (AF, GG, CMV) according to the AAI coding and classification system (Main et al., 2003). All transcripts were double-coded, blind to subject conditions. When disagreement arose, a third independent coder categorized the relevant transcript and, following discussion between all three coders, a final agreement was reached.

#### Complex Trauma Questionnaire (ComplexTQ; Maggiora Vergano et al., 2015)

The ComplexTQ is a 70-item scale for the retrospective assessment of multi-type maltreatment, measuring lack of care (physical and emotional neglect), abuse (psychological, physical and sexual abuse) and other traumatic experiences, such as rejection, role reversal, exposure to domestic violence, separation and loss. The instrument is available in two different versions: clinician-report and self-report. The questionnaire assesses adverse experiences from childhood (to the age of 14) involving maternal, paternal and other attachment figures, separately. In the current study, the clinician-report version was used and applied to the AAI transcripts. The questionnaire requires approximately 15 to 20 min to complete, depending on the length of the interview transcript. Scores for the presence and frequency of traumatic experiences in each domain are automatically provided by the software.

Concerning the analyses of the current study, for the attribution of attachment categories we used the scoring system of Main et al. (2003), which takes in account specific cut-offs for the state of mind scales. We considered these scales also for comparisons between groups and subgroups, to identify the prevalent representational characteristics of the participants. With regard to experience scales, we took in account only the Loving scale, which was identified as a protective factor against traumas (see Murphy et al., 2014). Other experience scales are explored in a thorough way by the ComplexTQ. Due to the large number of *t*-test comparisons, we performed a multiple testing correction using the Benjamini–Hochberg (BH) method (Benjamini and Hochberg, 1995) to adjust the *p*-values into false discovery rate (FDR).

## Results

### Attachment Classifications

Analysis of the 4-way distribution of AAI transcripts highlighted that, within the control group, 61% were classified as F, 19% were classified as Ds, 7% were classified as E and 13% were classified as U. With regard to the GD participants, 27% were classified as F, 14% were classified as Ds, 13% were classified as E and 46% were classified as U. Focusing on the disorganized portion of the GD sample, 19% were classified as U, 17% were classified as CC and 10% were classified as both U and CC. Despite the heterogeneity of the GD persons’ disorganized profiles, analysis of the standardized residuals highlighted an overrepresentation of CC (*sr* = 3.4) and U/CC (*sr* = 2.7) classifications.

**Table 1** shows the distribution of AAI classifications for GD patients and controls within the dimensions of security–insecurity and organization–disorganization. The GD patients’ distribution differed significantly from that of the controls on both comparisons. In the security–insecurity comparison (χ^2^[1, *N* = 218] = 24.347, *p* = 0.000, effect size φ = 1.65), there was an underrepresentation of F classifications (*sr* = -2.7) and an overrepresentation of insecure categories (*sr* = 2.5). Regarding the distribution of organized (F, Ds, and E) and disorganized (U and CC) classifications [*χ^2^*(1, *N* = 218) = 29.810, *p* = 0.000, effect size φ = 2.01], in the GD sample, disorganized states of mind were overrepresented (*sr* = 3.5) and, to a lesser extent, fewer organized attachments (*sr* = -2.2) than expected were found.

**Table 1:** Adult attachment interview (AAI) classifications distribution (secure vs. insecure and organized vs. disorganized) for GD patients and controls.

	Controls	GD patients
	(*n* = 123)	(*n* = 95)
Secure (F)	75 (61%)	26 (27%)
	(*sr* = 2.4)	(*sr* = -2.7)
Insecure (Ds, E, U, CC)	48 (39%)	69 (73%)
	(*sr* = -2.2)	(*sr* = 2.5)
Organized (F, Ds, E)	107 (87%)	51 (54%)
	(*sr* = 1.9)	(*sr* = -2.2)
Disorganized (U, CC)	16 (13%)	44 (46%)
	(*sr* = -3.1)	(*sr* = 3.5)

*F, free/autonomous; Ds, dismissing; E, entangled/preoccupied; U, unresolved/ disorganized; CC, cannot classify; *sr*, standardized residuals.*

We then compared the GD group to normative and combined clinical samples from international (Bakermans-Kranenburg and van IJzendoorn, 2009) and national meta-analyses (Cassibba et al., 2013), with reference to the 4-way AAI distribution and the organized vs. disorganized classifications (**Table 2**). Analyses of the 4-way distribution showed that our GD group differed significantly from normative samples – both international (χ^2^[3, *N* = 788] = 45.503, *p* = 0.000, effect size Cramer’s *V* = 0.94) and Italian (χ^2^[3, *N* = 937] = 119.800, *p* = 0.000, effect size Cramer’s *V* = 2.26), and, to a lesser extent, to the Italian clinical sample (*χ^2^*[3, *N* = 355] = 14.347, *p* = 0.002, effect size Cramer’s *V* = 0.44) – whereas no difference was found between our GD group and the international clinical sample. As to the comparison of organized and disorganized AAI classifications, the GD group was strongly significantly different from normative samples – both international (χ^2^[1, *N* = 788] = 39.085, *p* = 0.000, effect size φ = 1.39) and Italian (χ^2^[1, *N* = 937] = 115.369, *p* = 0.000, effect size φ = 3.77) – although it showed no differences relative to clinical samples.

**Table 2:** Comparison with literature data on AAI classifications (Bakermans-Kranenburg and van IJzendoorn, 2009^∗^; Cassibba et al., 2013^∗∗^).

	GD (*n* = 95)	Normative sample^∗^ (*n* = 693)	Clinical sample^∗^ (*n* = 1854)	Normative sample^∗∗^ (*n* = 842)	Clinical sample^∗∗^ (*n* = 260)
F	26 (27%)	392 (56%)	426 (21%)	508 (60%)	43 (17%)
Ds	13 (14%)	112 (16%)	389 (23%)	172 (21%)	79 (30%)
E	12 (13%)	63 (9%)	241 (13%)	92 (11%)	44 (17%)
U/CC	44 (46%)	126 (18%)	797 (43%)	70 (8%)	94 (36%)
Organized	51 (54%)	567 (82%)	1056 (57%)	772 (92%)	166 (64%)
Disorganized	44 (46%)	126 (18%)	797 (43%)	70 (8%)	94 (36%)

*F, free/autonomous; Ds, dismissing; E, entangled/preoccupied; U, unresolved/disorganized; CC, cannot classify.*

#### AAI State of Mind Scales

As mentioned above, we opted to explore the AAI state of mind scales for Idealization, Derogation and Anger. **Table 3** shows the mean scores of the three scales for the two groups. In GD patients, after the BH correction, only Anger toward fathers resulted significantly higher, relative to the control group.

**Table 3:** Mean scores (and standard deviations) of AAI state of mind scales for GD patients and controls.

		Controls (*n* = 123)	GD patients (*n* = 95)	*t* (*df* = 216)	Effect size (Cohen’s d)^a^	*p*	FDR^b^
Idealization	Mothers	2.77 (1.70)	3.29 (2.08)	–1.963	0.28	<0.05	n.s. (0.102)
	Fathers	2.26 (1.64)	2.02 (1.73)	1.446	–	n.s.	n.s.
Derogation	Mothers	1.25 (0.80)	1.34 (1.24)	–0.667	–	n.s.	n.s.
	Fathers	1.25 (0.80)	1.70 (1.82)	–2.214	0.33	<0.05	n.s. (0.086)
Anger	Mothers	1.53 (0.96)	1.50 (1.14)	0.229	–	n.s.	n.s.
	Fathers	1.40 (0.89)	1.97 (1.55)	–3.177	0.47	<0.05	0.011

*^*a*^Effect sizes Cohen’s *d*: 0.80 or higher is a large effect size, 0.50–0.79 a medium effect size and 0.20–0.49 small. Effect sizes less than 0.20 are negligible (Cohen, 1988). ^*b*^*P*-values were adjusted into false discovery rate (FDR) using the Benjamini–Hochberg (BH) method (Benjamini and Hochberg, 1995)*

#### *Trans* Women vs. *Trans* Men

With regard to the comparison between *trans* women and *trans* men in the GD sample, the 4-way AAI distribution, the distribution of secure vs. insecure AAI classifications and the distribution of organized vs. disorganized AAI classifications showed no significant differences. Concerning the AAI state of mind scales, the only significant difference between the two subgroups was observed with regard to Idealization (*t*[2.618, *p* = 0.012, *df* = 92]), showing that *trans* women idealized their mothers to a greater extent.

### History of Complex Trauma

#### Trauma Occurrence

Presence of a traumatic history was evaluated with the ComplexTQ, which considered participants’ experiences of many forms of relational trauma to the age of 14, within primary relationships. Results showed that 10% of GD participants had not experienced any early adversity, 13% had experienced one form of trauma, 8% had experienced two forms, 13% had experienced three forms and 56% had experienced four or more forms. In the control group, 30% of participants had not experienced any form of trauma, 37% had experienced one form of trauma, 16% had experienced two forms, 9% had experienced three forms and 7% had experienced four or more forms.

**Table 4** shows the distribution of individuals (GD patients and controls) with experience of fewer than four traumatic forms against those who were polyvictimised, showing experience of four or more forms of trauma. We opted for this grouping, following the examples of Finkelhor et al. (2007) and Murphy et al. (2014), who identified the co-occurrence of four or more forms of adversity as a marker of high traumatisation. The percentage of those who had experienced fewer than four forms of trauma was lower in the GD group (44%) than the control group (93%). The distribution in our sample strongly differed from that of the control group [χ^2^(1, *N* = 218) = 61.881, *p* = 0.000, effect size φ = 4.19], showing a robust overrepresentation of polyvictimisation (*sr* = 5.0) and an underrepresentation of fewer than four experienced forms of trauma (*sr* = -3.2).

**Table 4:** Occurrence of trauma (<4 vs. ≥4) for GD patients and controls.

	Controls	GD patients
	(*n* = 123)	(*n* = 95)
<4	114 (93%)	42 (44%)
	(*sr* = 2.8)	(*sr* = -3.2)
≥4	9 (7%)	53 (56%)
	(*sr* = -4.4)	(*sr* = 5.0)

*<4 = individuals with experiences of fewer than four typologies of trauma; ≥4 = individuals with experiences of four or more typologies of trauma (polyvictimisation).*

We also compared our GD group with the clinical sample of Maggiora Vergano et al. (2015), which was composed of individuals with dissociative and personality disorders. No significant differences were found between our group and the clinical sample, even when the occurrence of types of adversities were merged (<4 = absence vs. ≥4 = presence of polyvictimisation).

#### Trauma Frequency

For the comparison of our sample and controls regarding the frequency of experiences of various forms of trauma, the reader is referred to the following subsection, where the comparison is broken down according to gender.

The comparison with literature data (Maggiora Vergano et al., 2015) concerning the frequency observed in all ComplexTQ scales is shown in **Table 5**. In our sample, also after the BH correction, significantly lower scores were found on the following scales, all regarding the relationship with the mother: Neglect and Reject, and, to a lesser extent, Psychological Abuse, Physical Abuse and Witnessing Domestic Violence. There were no significant differences between the two samples for the Loss scales.

**Table 5:** Comparison of our GD sample and literature data (Maggiora Vergano et al., 2015^∗^) on frequency of trauma.

		GD (*n = 95*)	Clinical sample^∗^ (*n = 56*)	*t* (*df* = 149)	Effect size (Cohen’s *d*)	*p*	FDR
Neglect	Mothers	1.50 (0.65)	2.03 (0.66)	–4.768	0.81	<0.001	0.000
	Fathers	2.12 (0.92)	2.07 (0.76)	0.364	–	n.s.	n.s.
Reject	Mothers	1.33 (0.46)	1.75 (0.62)	–4.366	0.80	<0.001	0.000
	Fathers	1.65 (0.65)	1.64 (0.56)	0.059	–	n.s.	n.s.
Role reversal	Mothers	1.30 (0.42)	1.39 (0.62)	–0.990	–	n.s.	n.s.
	Fathers	1.05 (0.18)	1.12 (0.34)	–1.428	–	n.s.	n.s.
Psychological abuse	Mothers	1.11 (0.16)	1.22 (0.24)	–3.087	0.57	<0.05	0.015
	Fathers	1.15 (0.22)	1.18 (0.21)	–0.758	–	n.s.	n.s.
Physical abuse	Mothers	1.16 (0.29)	1.39 (0.54)	–2.979	0.57	<0.05	0.016
	Fathers	1.24 (0.43)	1.32 (0.49)	–1.063	–	n.s.	n.s.
Sexual Abuse	Mothers	1.02 (0.13)	1.04 (0.18)	–0.851	–	n.s.	n.s.
	Fathers	1.01 (0.08)	1.03 (0.15)	–1.165	–	n.s.	n.s.
	Other figures	1.09 (0.29)	1.06 (0.22)	0.584	–	n.s.	n.s.
Domestic violence	Mothers	1.21 (0.48)	1.46 (0.62)	–2.586	0.47	<0.05	0.021
	Fathers	1.27 (0.59)	1.46 (0.62)	–1.862	–	n.s.	n.s.
Separations	Mothers	1.20 (0.58)	1.29 (0.62)	–0.814	–	n.s.	n.s.
	Fathers	1.56 (0.90)	1.75 (0.89)	–1.243	–	n.s.	n.s.

### GD vs. Control Subgroups

We first compared trauma frequency between *trans* women and male controls, and subsequently between *trans* men and female controls.

The intensity of different traumatic experiences suffered by the GD and control subgroups, as yielded by the ComplexTQ scales, is shown in **Tables 6A, B**. Regarding the comparison between *trans* women and male controls, *trans* women resulted to have, following the BH correction, significantly higher scores on the following scales: father’s Reject, Physical and Psychological Abuse, and Neglect from both parents. Fathers’ Neglect and Reject yielded the largest effect sizes. Difference was significant with respect to father’s Loss, with higher scores for *trans* women [Fisher’s exact test: (*N* = 135), *p* = 0.002].

**Table 6A:** Comparison of subgroups (male controls vs. *trans* women) with regard to frequency of trauma.

		Male controls (*n* = 61)	*Trans* women (*n* = 74)	*t* (*df* = 133)	Effect size (Cohen’s *d*)	*p*	FDR
Neglect	Mothers	1.23 (0.34)	1.49 (0.66)	–2.963	0.48	<0.05	0.021
	Fathers	1.39 (0.38)	2.08 (0.89)	–6.076	0.98	<0.001	0.000
Reject	Mothers	1.16 (0.38)	1.32 (0.47)	–2.097	0.37	<0.05	n.s. (0.080)
	Fathers	1.14 (0.25)	1.66 (0.68)	–6.192	0.98	<0.001	0.000
Role reversal	Mothers	1.15 (0.35)	1.28 (0.41)	–1.873	–	n.s.	n.s.
	Fathers	1.01 (0.05)	1.06 (0.19)	–2.182	0.35	<0.05	n.s. (0.078)
Psychological abuse	Mothers	1.09 (0.18)	1.09 (0.14)	–0.073	–	n.s.	n.s.
	Fathers	1.07 (0.13)	1.14 (0.20)	–2.509	0.41	<0.05	0.045
Physical abuse	Mothers	1.13 (0.29)	1.16 (0.28)	–0.521	–	n.s.	n.s.
	Fathers	1.09 (0.23)	1.23 (0.40)	–2.573	0.42	<0.05	0.045
Sexual abuse	Mothers	1.00 (0.00)	1.02 (0.14)	–1.375	–	n.s.	n.s.
	Fathers	1.00 (0.00)	1.01 (.09)	–0.907	–	n.s.	n.s.
	Other figures	1.01 (0.06)	1.07 (0.26)	–1.854	–	n.s.	n.s.
Domestic violence	Mothers	1.13 (0.28)	1.18 (0.44)	–0.672	–	n.s.	n.s.
	Fathers	1.12 (0.26)	1.26 (0.57)	–1.829	–	n.s.	n.s.
Separations	Mothers	1.06 (0.24)	1.23 (0.62)	–2.178	0.35	<0.05	n.s. (0.078)
	Fathers	1.30 (0.56)	1.54 (0.91)	–1.858	–	n.s.	n.s.

**Table 6B:** Comparison of subgroups (female controls vs. *trans* men) with regard to frequency of trauma.

		Female controls (*n* = 62)	*Trans* men (*n* = 21)	*t* (*df* = 81)	*Effect size* (Cohen’s *d*)	*p*	FDR
Neglect	Mothers	1.31 (0.44)	1.54 (0.64)	–1.565	–	n.s.	n.s.
	Fathers	1.27 (0.38)	2.28 (1.06)	–4.276	1.62	<0.01	0.005
Reject	Mothers	1.19 (0.29)	1.36 (0.43)	–2.040	0.51	<0.05	n.s. (0.122)
	Fathers	1.14 (0.36)	1.60 (0.56)	–3.521	1.10	<0.05	0.014
Role reversal	Mothers	1.10 (0.23)	1.37 (0.48)	–2.512	0.87	<0.05	n.s. (0.065)
	Fathers	1.04 (0.10)	1.02 (0.09)	0.716	–	n.s.	n.s
Psychological abuse	Mothers	1.04 (0.08)	1.18 (0.20)	–3.166	1.15	<0.05	0.025
	Fathers	1.04 (0.08)	1.19 (0.27)	–2.451	0.99	<0.05	n.s. (0.110)
Physical abuse	Mothers	1.06 (0.13)	1.15 (0.31)	–1.437	–	n.s.	n.s
	Fathers	1.03 (0.09)	1.28 (0.56)	–2.066	–	n.s.	n.s.
Sexual abuse	Mothers	1.00 (0.00)	1.02 (0.09)	–1.000	–	n.s.	n.s.
	Fathers	1.00 (0.00)	1.00 (0.00)	–	–	n.s.	n.s.
	Other figures	1.01 (0.04)	1.16 (0.37)	–1.835	–	n.s.	n.s.
Domestic violence	Mothers	1.09 (0.22)	1.33 (0.61)	–1.802	–	n.s.	n.s.
	Fathers	1.09 (0.22)	1.31 (0.64)	–1.547	–	n.s.	n.s.
Separations	Mothers	1.06 (0.24)	1.12 (0.38)	–0.875	–	n.s.	n.s.
	Fathers	1.09 (0.33)	1.64 (0.90)	–2.769	1.04	<0.05	0.048

In the comparison between *trans* men and female controls, after the BH correction, yielding large effect sizes, *trans* men showed significantly higher scores on the following scales: fathers’ Neglect, Reject and Separation, and mothers’ Psychological Abuse. Moreover, higher scores for *trans* men were found with respect to the Loss of other figures [χ^2^(1, *N* = 83) = 14.512, *p* = 0.000, effect size φ = 1.59].

#### *Trans* Women vs. *Trans* Men

With reference to the comparison within the GD sample between *trans* women and *trans* men, no significant differences were observed in the occurrence of trauma, the classification of polyvictimisation or the frequency of traumatic experiences, as examined through each ComplexTQ scale.

### Caregiving

To explore loving parental caregiving, we considered the mean scores of the AAI Loving scale. A significant difference was observed between GD and controls with respect to the father’s relationship [GD = 3.11(1.59), Controls = 4.16(1.36); *t*(4.979, *p* < 0.001, *df* = 206, effect size Cohen’s *d* = 0.70)], showing the poor emotional support and availability of fathers of GD persons. No significant difference was found in the comparisons between control males and *trans* women and between control females and *trans* men, nor between *trans* women and *trans* men.

## Discussion

In the current study, we explored the attachment representations and narratives and early relational history of a sample of GD adults at two levels of analysis. The first level considered the entire GD sample and compared this sample with controls and other samples from the literature; the second level narrowed the investigation to GD subgroups, comparing attachment and early relational experiences between *trans* women and *trans* men and experiences of trauma between *trans* women and male controls and between *trans* men and female controls. In the current section, summaries of the main findings are presented for the reader’s convenience.

Concerning the first level of inquiry (into attachment), it is noteworthy to underline that almost half of our sample (46%) was marked by a high percentage of U and CC classifications, indicating a prevalence of insecurity. In the control group, only one form of disorganization was found: the U profile with regard to loss, due in most cases to recent deaths. In contrast, the GD sample showed all three forms of disorganized profiles (19% U, 17% CC and 10% U/CC).

In this regard, the results showed that our GD sample was significantly aligned with Italian (Cassibba et al., 2013) and international clinical populations (Bakermans-Kranenburg and van IJzendoorn, 2009), which have been found to reach combined U and CC percentages of between 36 and 43%. It is interesting to note that the literature data highlights high percentages of disorganization in clinical samples (Allen et al., 1996; West et al., 2001; Steele et al., 2003; Agrawal et al., 2004; Stovall-McClough and Cloitre, 2006; Harari et al., 2007; Ivarsson, 2008; Farina et al., 2014; Murphy et al., 2014) and high psychosocial risk (Stalker and Davies, 1995; Lyons-Ruth et al., 2003; Crowell and Hauser, 2008). Specifically, with respect to GD samples, the high occurrence of U and CC in our group confirmed the association between disorganized patterns and transsexualism that has been found in previous studies (Cook, 1999; Vitelli and Riccardi, 2010; Lingiardi et al., 2017). Moreover, the heterogeneity of disorganized profiles that defined our GD sample denoted the presence of both a local breakdown in discourse strategies regarding loss or abuse and a global disruption of attachment strategies along the entire transcript. Many of the participants’ attachment narratives were characterized by a lack of autobiographical coherence and integration, revealing difficulties in connecting the past and present elements of one’s personal history, depicting balanced portraits of caregiving figures and giving importance to attachment experiences. As was found in the clinical populations cited above, these deficits could be attributable to a harsh and careless caregiving environment during childhood; such an environment may have undermined our participants’ personal sense of safety.

Significant differences were also found in specific AAI state of mind scales: our GD group showed higher Anger toward fathers and, to a lesser extent (with smaller effect sizes), Derogation toward fathers and Idealization toward mothers, relative to controls.

With regard to these comparisons regarding state of mind scales, our aim was not to typify our individuals as belonging to specific attachment classifications, rather to compare representational ‘stances’ between groups and subgroups. The emerged polarized representations of parental figures are worthy of notice, since they add another aspect of unbalance to the participants’ attachment narratives regarding representations of their parents. As mentioned above, psychological and psychoanalytical theories have mostly seen family dysfunctions as etiological factors of transsexual conditions. In contrast, we took an agnostic approach to causation, considering insecure and disorganized attachment patterns – as well as adversity in early relationships – as potential concurrent risk factors for GD persons’ psychological development. Despite resounding psychoanalytical etiological accounts such as Coates’ (Coates and Person, 1985) focus on *trans* children’s separation anxiety from their mother and their fantasized connection to her and Stoller’s (1968; 1985) remark about fathers’ absence and emotional unavailability, these findings should be interpreted cautiously, especially those regarding Idealization and Derogation, that resulted not significant following the Bergamini–Hochberg correction, and should not be understood to suggest causation. We do not consider these states of mind (Idealization of mothers and Anger and Derogation toward fathers) signals of ancient psychological identifications that form gender identity; rather, we see them as opposing representations of acceptance and non-acceptance of parental figures.

Considering the second major focus of the study, relating to personal histories of complex trauma, the presence and intensity of multiple forms of early relational trauma were investigated. In more than half of *trans* participants (56%), we observed a co-occurrence of multi-type maltreatments during childhood, leading to polyvictimisation (Finkelhor et al., 2007, 2011). Relative to controls, GD participants had experience of significantly more traumatic forms, including both active trauma and severe neglect. Considering the wide range of traumatic experiences found in this sample, these findings assuredly indicate the presence of complex trauma, highlighting differences in the parental couple. We discuss the parent–child relationship in more detail below, in our evaluation of the comparisons between subgroups.

In our comparison of the GD group to a sample of highly traumatized individuals with dissociative and personality disorders (Maggiora Vergano et al., 2015), we found similar levels of polyvictimisation. However, there were significant differences in the frequency of different types of trauma in the relationship with mothers: GD individuals were less neglected, rejected and psychologically and physically abused, and witnessed less domestic violence involving their mothers compared to the clinical sample. This outcome highlights the more supportive role that mothers might have had in our sample; in light of a comparable frequency of traumatic experiences, mothers of GD youth seem to have provided better backup. In contrast, relationships with fathers were similarly dysfunctional and compromised in both samples.

In general, the results regarding trauma confirm and extend previous findings on risk for maltreatment and abuse for *trans* youth (e.g., Nuttbrock et al., 2009, 2010; Bandini et al., 2011). Our participants were exposed to several early relational forms of trauma and victimization, from multiple sources. This outcome highlights the degree of compromise in their caregiving environment and may explain the high frequency of attachment disorganization in the sample.

In order to obtain a better understanding of the quality and nature of GD participants’ relationships with primary figures, we also explored their positive experiences of loving caregiving. We found that fathers of GD participants were less accepting, available and supportive compared to fathers of controls. There were no significant differences in mothers between groups. We should consider how, beyond the connections between traumatic experiences and GD, the complicated development of a gender atypical identity may have been further hindered by a lack of support and exposure to trauma within the family, especially from fathers. This may have led GD persons to separate and dis-integrate their representations, memories and experiences. Relationships with mothers seemed less traumatic, but, compared to controls, mothers of our GD participants were more idealized. In this regard, we must consider a more general aspect: as attested by the literature and in line with the social attitudes and culture at the time of our participants’ childhood, when children need care due to illness or distress, mothers tend to assume the major responsibility for this care, whereas fathers tend to carry on with duties related to the maintenance of the household and are less involved in affectively supporting the child (e.g., Mattson and Weisberg, 1970; Frank et al., 1986). Moreover, fathers are more likely to expect their children to adhere to cultural stereotypes (e.g., Langlois and Downs, 1980). With respect to our GD sample, and supported by Wren’s (2002) research on transgender adolescents, the participants’ fathers may have cut themselves off from emotional issues, while their mothers may have maintained a better relationship with their children and been – at least by all appearances – more accepting. High scores on Idealization of mothers may suggest that this positive representation was, in most cases, generalized and skin-deep, as it was unsupported by recounted episodes.

Following this exploration, we engaged in a deeper level of analysis, splitting our sample into two subgroups of *trans* women and *trans* men and the control group into males and females.

Regarding attachment patterns, no significant difference was observed in the comparison between GD subgroups. This highlights the homogeneity in our sample with regard to the distribution of attachment classifications. Therefore, dysfunctionality in attachment may not be specifically linked with experienced gender but, more generally, with the fact that *trans* people are a group at risk. Moreover, as Bakermans-Kranenburg and van IJzendoorn (2009) state, no significant gender difference has been found in numerous studies using the Adult Attachment Interview.

Concerning the AAI scales, *trans* women showed greater Idealization of mothers than did *trans* men. Thus, the greater Idealization of mothers in our sample relative to controls could be largely attributable to the *trans* women subsample. We discuss this finding further, below.

Regarding trauma history, whilst no difference emerged from the comparison within the GD group, interesting findings emerged from comparisons of the GD sample and controls, when divided according to natal gender (*trans* women vs. control males; *trans* men vs. control females). *Trans* women, compared to control males: (1) were more neglected by both parents; (2) had more involving, rejecting and physically and psychologically abusive fathers; and (3) suffered more frequently from an early loss of the father. On the other hand, *trans* men, compared to female controls: (1) were more often victims of intensive rejection, neglect and early separation from fathers; (2) had more psychologically abusive mothers; and (3) prematurely experienced more losses of close relatives and friends.

The overall findings of this deeper level of enquiry highlight some interesting aspects of parental aversive relationships and representations in the two GD subgroups. Overall, both subgroups suffered from severe neglect, rejection and psychological abuse. The results of the *trans* women are particularly interesting because they disprove the classical psychoanalytic view of female transsexuals’ fathers only as absent figures. In our sample, the *trans* women’s fathers, although strongly neglecting and rejecting their children, were also present, involving and psychologically abusive, and their mothers were not blissfully symbiotic – they too neglected their children, though to a lesser degree than the fathers. For the *trans* women, idealization of mothers could be interpreted as idealization of the feminine figure or feminine aspects they felt were their own.^2^ Moreover, it is interesting to note that regarding psychological abuse the comparison between *trans* men and female controls presented a reversed finding: the main psychologically abusive figure in the *trans* men’s parental relationships was the mother, and not the father.

These outcomes have several implications for clinical practice, family support and education. Assessment and clinical management of GD patients are often provided within multidisciplinary gender clinics. In these centers, patients are followed by psychiatrists, pediatricians, psychologists, and psychotherapists, working side by side with endocrinologists and surgeons. Within psychological assessment and support, we believe that attachment and trauma investigation could play a crucial role in bringing to light conflicts and defenses that may interfere with a free exploration of gender identity. Such an exploration might suffer from the negative impact of early relationships with parental figures. Interiorisation of early experiences may pollute a profound sense of gender identity and expose *trans* persons to emotional vulnerability during the delicate course of their transition path. Psychologists and psychotherapists should help GD patients to integrate traumatic memories in order to enhance their resilience and comfort in their gender identity expression. Professionals should be trained in and informed on GD and its traumatic correlates in order to offer patients and families qualified and ethical support. Furthermore, knowledge on GD should be improved among families, schools and communities in order to promote social acceptance, reduce stigma and defend against dangerous risk factors for *trans* persons’ psychological wellbeing. This need is particularly urgent in Italy, where knowledge and information on GD seem very scarce in many contexts (Fisher et al., 2016).

The present study is not without limitations. First, the sample size was relatively small and thus generalization of the results should be made with caution; however, small sample sizes are common in Italian studies with GD patients (the number of referrals in Italian gender clinics is much smaller than that of other European nations or the United States). Second, findings should be generalized cautiously also by reason of the lack of a control group paired for age, numerosity and education. Finally, in this study, trauma history was assessed with the clinician-report ComplexTQ, as applied to AAI transcripts; therefore, trauma was not evaluated from the attachment measure through an independent source. However, as Bailey et al. (2007) claim, AAI can elicit more reports of physical abuse than other trauma interviews, because it frames questions about adversity within the broader context of childhood familial experiences and relationships, facilitating the recall of more elusive traumatic forms such as neglect or emotional abuse. Future research should seek to expand these findings in studies with paired control samples and in longitudinal designs that assess individuals during the course of gender transitions, prior and after hormone therapy and gender reassignment surgery. Research on early traumatic experiences and mental states with regard to attachment in gender variant individuals should benefit also of studies that assess trauma and attachment from independent sources.

As mentioned above, the aim of the study was not to capture the causal relationships between trauma, attachment and GD. Rather, we considered both trauma and GD multi-dimensional constructs requiring no reductive or short-sighted attempts at explanation. Further research, however – likely using longitudinal designs – might explore more deeply the influence of early traumatic experiences and disorganized attachment representations on the development of a non-conforming gender identity.

## Author Contributions

GG: Conception of the work, data collection, data analysis and interpretation, drafting the article. RV: Conception of the work, data collection, data analysis and interpretation. CM: Data analysis and interpretation, drafting the article. AF: Data collection, data analysis and interpretation, drafting the article. LC: Data collection. VL: Conception of the work, data analysis and interpretation, critical revision of the article. AS: Conception of the work, critical revision of the article, final approval of the version to be published.

## Conflict of Interest Statement

The authors declare that the research was conducted in the absence of any commercial or financial relationships that could be construed as a potential conflict of interest.

## Footnotes

1 In this paper, following Serano’s (2007) recommendations for less discriminatory terminology, we favor the forms “*trans* woman" (for natal males) and “*trans* man" (for natal females) over other widely used forms, such as “male-to-female" (MtF) or “female-to-male" (FtM). In this respect, we also wish to thank MD Jack Drescher (member of the DSM-5 Workgroup on Sexual and Gender Identity Disorders and the ICD-11 Working Group on the Classification of Sexual Disorders and Sexual Health) for his advice.

2 In this regard, it is interesting to note that, in many cases, the adjectives chosen by *trans* women to describe their mothers were generic and stereotyped (e.g., “all-mothery,” “mother love of my life,” “we were all one,” “good mother”) and often related to physical aspects (“beautiful,” “seductive,” “all men liked her”).
